# The Efficacy of Machine-Learning-Supported Smart System for Heart Disease Prediction

**DOI:** 10.3390/healthcare10061137

**Published:** 2022-06-18

**Authors:** Nurul Absar, Emon Kumar Das, Shamsun Nahar Shoma, Mayeen Uddin Khandaker, Mahadi Hasan Miraz, M. R. I. Faruque, Nissren Tamam, Abdelmoneim Sulieman, Refat Khan Pathan

**Affiliations:** 1 Department of Computer Science and Engineering, BGC Trust University Bangladesh, Chittagong 4381, Bangladesh; nabsar@bgctub.ac.bd (N.A.); emondas457@gmail.com (E.K.D.); shomathetics@yahoo.com (S.N.S.); 2 Centre for Applied Physics and Radiation Technologies, School of Engineering and Technology, Sunway University, Petaling Jaya 47500, Selangor, Malaysia; 3 Department of General Educational Development, Faculty of Science and Information Technology, Daffodil International University, DIU Rd, Dhaka 1341, Bangladesh; 4 Department of Business Analytics, Sunway University, Petaling Jaya 47500, Selangor, Malaysia; mahadihm@sunway.edu.my; 5 Space Science Center, Universiti Kebangsaan Malaysia, Bangi 43600, Selangor, Malaysia; rashed@ukm.edu.my; 6 Department of Physics, College of Science, Princess Nourah Bint Abdulrahman University, Riyadh 11671, Saudi Arabia; nmtamam@pnu.edu.sa; 7 Department of Radiology and Medical Imaging, Prince Sattam Bin Abdulaziz University, Alkharj 11942, Saudi Arabia; a.sulieman@psau.edu.sa; 8 Department of Computing and Information Systems, School of Engineering and Technology, Sunway University, Petaling Jaya 47500, Selangor, Malaysia; refatkhan93@gmail.com

**Keywords:** decision tree, random forest, KNN, AdaBoost, heart disease, prediction, smart system

## Abstract

The disease may be an explicit status that negatively affects human health. Cardiopathy is one of the common deadly diseases that is attributed to unhealthy human habits compared to alternative diseases. With the help of machine learning (ML) algorithms, heart disease can be noticed in a short time as well as at a low cost. This study adopted four machine learning models, such as random forest (RF), decision tree (DT), AdaBoost (AB), and K-nearest neighbor (KNN), to detect heart disease. A generalized algorithm was constructed to analyze the strength of the relevant factors that contribute to heart disease prediction. The models were evaluated using the datasets Cleveland, Hungary, Switzerland, and Long Beach (CHSLB), and all were collected from Kaggle. Based on the CHSLB dataset, RF, DT, AB, and KNN models predicted an accuracy of 99.03%, 96.10%, 100%, and 100%, respectively. In the case of a single (Cleveland) dataset, only two models, namely RF and KNN, show good accuracy of 93.437% and 97.83%, respectively. Finally, the study used Streamlit, an internet-based cloud hosting platform, to develop a computer-aided smart system for disease prediction. It is expected that the proposed tool together with the ML algorithm will play a key role in diagnosing heart diseases in a very convenient manner. Above all, the study has made a substantial contribution to the computation of strength scores with significant predictors in the prognosis of heart disease.

## 1. Introduction

According to the World Health Organization, cardiovascular diseases (CVDs) cause the death of 17.9 million people each year, making them the leading cause of death worldwide [1]. Several reasons including overweight and obesity, hypertension, hyperglycemia, high alcohol intake, etc., are identified as the main risk factors for this disease [1]. Although some risk factors are controllable, and various metabolic symptoms can be used for predicting heart conditions, physicians nevertheless find it difficult to correctly and quickly diagnose cardiac disease based on risk factors [2]. In fact, the prognosis of CVDs is complicated by their clinical symptoms, which are impacted by various functional and pathologic appearances. Various computational techniques are employed in different medical prognoses of coronary heart disease (CHD) symptoms [3,4,5,6,7,8,9,10,11] such as hyperlipidemia, myocardial infarction, angina pectoris, etc. [12,13,14]. Medical experts use electrocardiography, sonography, angiography, and blood test to diagnose CHD. Although it is difficult to diagnose CHD in the early stages of the illness [15,16,17,18], early detection is crucial for effective treatment [19,20,21,22,23]. 

Many studies on clinical decision-support systems [20] have been undertaken to overcome these difficulties by utilizing diverse techniques such as data mining and machine learning [21,22,23,24,25]. In line with medical diagnostics, a variety of data mining approaches such as neural networks [26,27], hybridized rough sets [28,29,30], and fuzzy learning vector quantization networks [31] have been developed. The medical applications of these techniques have used association rules [32], principal component analysis, and radial basis function neural network [33]. The neural network (NN) is the most commonly utilized technology to improve performance accuracy in CHD prediction [19,34,35,36,37,38]. Without prior domain knowledge of CHD, NN is good at generalizing data. NN also enables the discovery of novel patterns and information relevant to CHD by evaluating complex data [39,40,41]. However, anomaly detection from massive datasets has recently been the subject of specific research [42,43,44,45]. Therefore, developing an intelligent CHD forecast model for early-stage disease prediction at a cheap cost is crucial. In fact, machine learning techniques with various classifiers/models can be utilized to predict such diseases based on the existing data.

Data processing with machine learning classifiers may play a significant role in the prognostication of heart conditions [46]. In recent times, several studies (presented in the next section) have been conducted for this purpose. All of these studies revealed that the use of computerized medical decision-support systems is a viable method for assisting clinicians in making accurate and timely diagnoses of patients [47]. In this regard, more machine learning models need to be studied using various recent databases and used to obtain the best model for early-stage disease prediction at a low cost. Therefore, an attempt is made to bridge the experts’ knowledge and experience in order to create a system that equitably supports the diagnosis process.

The goal of this research is to use several computational intelligence techniques such as K-nearest neighbor (KNN), random forest (RF), decision tree (DT), and AdaBoost (AB) to predict cardiac illness through the internet and mobile apps. The KNN was chosen because it provides extremely precise predictions and can compete with the most accurate models. The distance measure determines how accurate the forecasts are. As a result, the KNN approach can be employed in applications where high accuracy is required. RF is a method that uses ensemble learning and is based on the bagging algorithm. DT is good at handling data and performs best with a linear pattern. It is capable of processing large amounts of data in a short time. It develops as many trees as feasible on a subset of the data, then merges all of the trees’ findings. On the other hand, instead of reducing variance, boosting reduces bias. In boosting, models are weighed based on their performance. That is why boosting is preferable to bagging. As a result, AdaBoost (AB) is best-suited for struggling samples. Our main aim is to improve the accuracy of the aforementioned ML models and then develop a computer-aided smart system to anticipate CHD sickness through an internet-based cloud hosting platform named Streamlit. It is anticipated that the proposed tool will play an essential role in identifying cardiac problems in a highly convenient manner. 

The rest of the paper is laid out in the following way: Section 2 shows other similar works. Section 3 explains the process flow of the work. Section 4 talks about the design and implementation of the study. Section 5 contains the experimental results and discussion, and Section 6 summarizes the study.

## 2. Related Work

A review of the literature shows that a range of ML techniques is utilized for disease prediction by many researchers worldwide. To predict cardiac disease, Ayon et al. [2] utilized several ML models such as SVM (support vector machine), DNN (deep neural network), DT (decision tree), NB (naïve Bayes), RF (random forest), LR (linear regression), and K-NN (k-nearest neighbor) on five-fold in the statlog dataset and obtained precision accuracies of LR (96.29%), SVM (97.41%), DNN (98.29%), DT (96.42%), NB (90.47%), RF (90.46%), and K-NN (96.42%). The authors also used the Cleveland dataset and obtained prediction accuracies of NB (91.18%), SVM (97.36%), DT (92.76%), RF (89.41%), K-NN (94.28%), DNN (94.39%), and LR (92.41%). In [48], the author proposed heart disease danger prediction based on LR, NN, Framingham risk score (FRS), and feature correlation analysis (FCA) and achieved accuracies of LR (86.11%), NN (87.04%), FRS (6.67%), and NN_FCA (87.63%) from the training set. Besides that, in the validation set, they obtained LR (80.32%), NN (81.09%), FRS (28.87%), and NN_FCA (82.51%) accuracy. In [46], the author studied hybrid machine learning techniques using NB, generalized linear model (GLM), logistic regression (LR), deep learning (DL), DT, RF, gradient boosted trees (GBT), SVM, and hybrid random forest linear model (HRFLM) to predict heart disease. The accuracy for these models are NB (75.8%), GLM (85.1%), LR (82.9%), DL (87.4%), RF (86.1%), GBT (78.3%), SVM (86.1%), and HRFLM (88.4%). In [49], the authors used an efficient hybrid algorithmic approach for heart disease prediction. They used the UCI Heart Disease Dataset and obtained accuracies of NB (88%), KNN (93%), and hybrid (97%). In [46], the author presented a method for diagnosing heart illness using ECG data that achieves excellent accuracy in a short time. They tested four classification methods: long–short-term memory (LSTM), dynamic temporal distortion (DTW), move-split-merge (MSM), and complexity invariant distance (CID). Among the various approaches, the LSTM unceasingly obtains a high accuracy of around 97%, without any preprocessing step. Furthermore, using a preprocessing technique (Symbolic Aggregate ApproXimation, SAX), the classification accuracy was reported to be 98.4%, and the reaction time is considerably faster than the approach adopted without preprocessing. Tülay and Özkan [50] examined the prediction of heart disease by using neural network with the Cleveland dataset. They tried to raise the reduction in representation dimensionality with major component analysis by diminishing the number of neurons in the input layer. They reported the highest accuracy of 95.55% using classification performance with principal component analysis (PCA). Purushottam et al. [51] presented an efficient heart disease prediction system using data mining. The authors used the Cleveland dataset and obtained the highest accuracy for the radial basis function (RBF) kernel (78.53%) and SVM (70.59%). Khaled [52] attempted to predict heart disease and classifiers’ sensitivity analysis. They used various classification algorithms to distinguish the classifiers’ actions in terms of the classification of the considered HD dataset, and then, a peculiarity-wrenching method was used to obtain the quality of the generated subsets and to evaluate the classification performance. This paper’s accuracy was KNN (99.70%), JRip (97.26%), and J48 (98.04%). The authors [53] proposed utilizing a convolutional neural network method to predict illness risk using organized and unstructured patient data. The created model achieves an accuracy of between 85 and 88%. In the [54], the authors suggested a model based on the K-means clustering method for detecting anomalies in the healthcare sector, with the best value of K assessed using the silhouette approach. They reported that the RF, SVM, and LR classifiers performed much better in the dataset without anomalies than those with anomaly instances. Kumar and Inbarani [54] discovered a procedure for recognizing coronary heart disease that combined classification strategies with particle swarm optimization (PSO). The method utilized short and relevant optimization to find the best characteristics. They used the outcome as input for machine learning techniques such as K-NN, multilayer perceptron (MLP), SVM, and backpropagation processes to classify the dataset. They acquired accuracies of 81.73%, 82.30%, 75.37%, and 91.94%, respectively. Rajathi and Radhamani [55] created a model combining KNN and ant colony optimization (ACO) strategies for coronary heart disease prediction and obtained an accuracy of 70.26% for four machine learning approaches [56]. Vineet et al. [57] focused on obtaining the greatest outcomes based on neural networks. Several models were created, their performance measurements were gathered, and then, the models’ results were compared against each other to determine the best possible result. The assessment of DNN was compared to other classifiers as part of the validation process. In this paper, they used SVM, naïve Bayes, KNN, and DNN, and the performed result was SVM (86.2%), NB (83.97), KNN (81.43%), and DNN (81.9%). Amin et al. [58] advocated for a hybrid paradigm in which the basic risk factors categorize the cardiac disease. They used two well-known technologies for their system: genetic algorithms and neural networks. Researchers initialized the weight of individual neurons on the neural networks that handle a genetic algorithm and universal optimization procedures. The study revealed that their model is fast compared to other models, with an accuracy of 89%. The authors of [59] represented a cardiopathy prediction way that utilizes a multilayer perceptron neural network. In a programmed manner, the NN accepts thirteen clinical selections as input and is trained by a backpropagation perception to predict the manner or inadequacy of heart problems in the patient with an accuracy of 98%. In [60], the authors performed machine pattern procedures, combined with a decision tree, approximation set, naïve Bayes, neural networks, and SVM and examined their exactitude and prediction and achieved an F-measure of 86.8%. They also proposed a replacement neural network (ANN) technique for categorizing arterial blood vessel stenting disease (CAS). In [61], planners presented various data processing and neural network classifier systems culturally appropriated to forecast heart condition likelihood. Additionally, it was shown that analyzing the hazard level of private exploitation procedures similar to DT, KNN, genetic algorithm (GA), and NB is high once used. They also introduced a computer-assisted decision network.

## 3. Methodology

**Figure 1:**
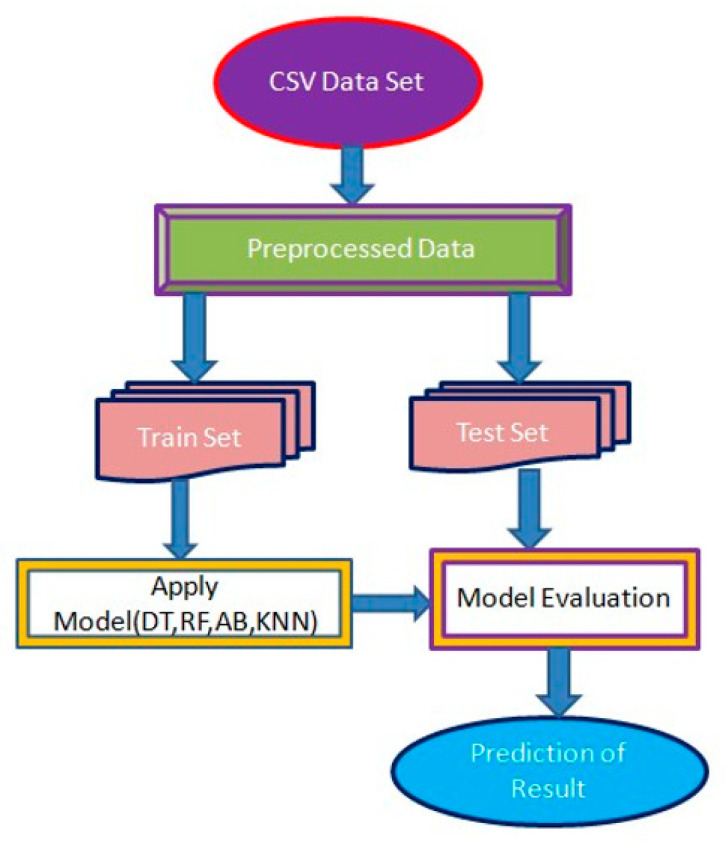
The system architecture of the present work.

The research model was evaluated using supervised learning techniques such as random forest and decision trees. Figure 1 shows a schematic illustration of the design of this study.

This new model was built using a brand-new batch of data. The researchers followed multiple steps to create the system, as shown in Algorithms 1 and 2.

**Algorithm 1:** Algorithms for the CHSLB dataset used in this study.**Input:** symptoms**Output:** predict heart disease present or not present1. If (the model has not been trained), then2. Dataset load;3. Correlation of data;4. Split x and y;5. Train (70%), test (30%);6. Load pre-trained model;7. Educate the model;8. Save the model that has been trained.11. Loads trained model if everything else fails;12. Validate the model using the test data set;13. Confusion metrics and plot graphs.

**Algorithm 2:** The algorithm for the Cleveland dataset used in this study.**Input:** symptoms**Output:** predict heart disease present or not present1. If (the model has not been trained), then2. Dataset load;3. Correlation of data;4. Check outliers;5. Remove outliers;6. Split x and y;7. Train (80%), test (20%);8. Load pre-trained model;9. Educate the model;10. Save the model that has been trained.11. Loads trained model if everything else fails;12. Validate the model using the test data set;13. Confusion metrics and plot graphs.

The overall performance of the pre-trained models is evaluated using four criteria: true positive = *TP*, true negative = *TN*, false positive = *FP*, and false negative = *FN*. The system’s performance is assessed by using the Equations (1)–(4)

(1) $$Accuracy=\frac{\left(TP+TN\right)}{\left(TP+TN+FP+FN\right)}$$

(2) $$Precision=\frac{TP}{\left(TP+FP\right)}$$

(3) $$Recall=\frac{TP}{\left(TP+FN\right)}$$

(4) $$F1\;Score=\frac{2\;*\;Precision\;*\;Recall}{Precision+Recall}$$

Considering that when the balance of the samples is adequately predicted, the class of matter is genuinely positive and in the case of the class of matter is a genuine negative, the balance of the samples is not adequately predicted. The dimension of units mislabeled as a class of interest is known as false positive. The fraction of samples mislabeled as non-class of interest is false negative [62].

## 4. Design and Implementation

### 4.1. Dataset

#### Data Collection

From the *Kaggle* database, the heart disease data were extracted from the Cleveland dataset [63]. Males and females are represented in patients’ datasets. The samples were split into 13 characteristics, with the class distribution being the 14th. In the collected dataset, 138 persons do not have heart disease, while 165 persons do. There are no missing data in this dataset.

The other data were extracted from four datasets: Cleveland, Hungary, Switzerland, and Long Beach (CHSLB) [64]. Patients’ datasets contain both males and females. There seem to be 1025 data in all, split into 13 characteristics, with the class distribution being the 14th attribute. Besides that, a total of 499 persons were healthy and heart-disease-free among the individuals studied, while the remaining 526 are sick. Furthermore, it indicates that there are no missing values. Likewise, data were obtained via the *Kaggle* database. Table 1 provides the data for both datasets.

### 4.2. Implementation of the System

The Python programming language was used to create the system, and it is still in use today. Matplotlib, Numpy, and Keras are the libraries utilized in this system.

### 4.3. Experimental Setup

Python 3.9.5 was used to carry out the experiment. The test was carried out on a single machine running Windows 10 pro (Lenovo, Intel (R) Core (TM) i3-7020U CPU, 2.30 GHz, RAM 4 GB).

### 4.4. Data Preprocessing

**Figure 2:**
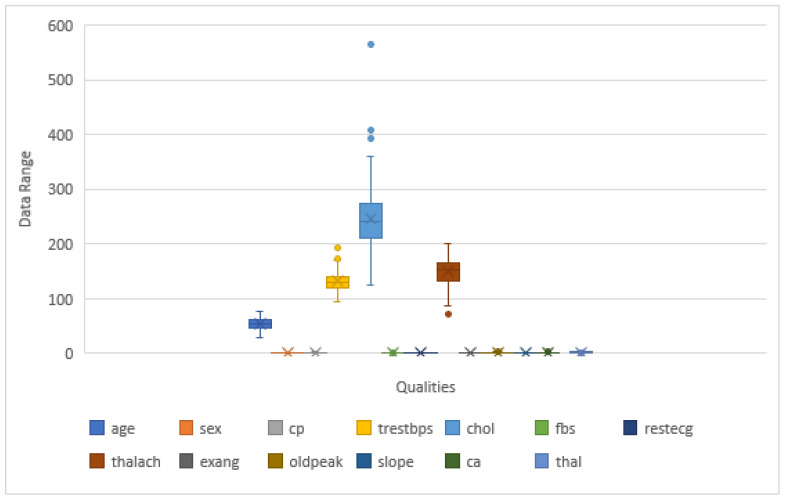
The outliers present in the Cleveland dataset.

**Figure 3:**
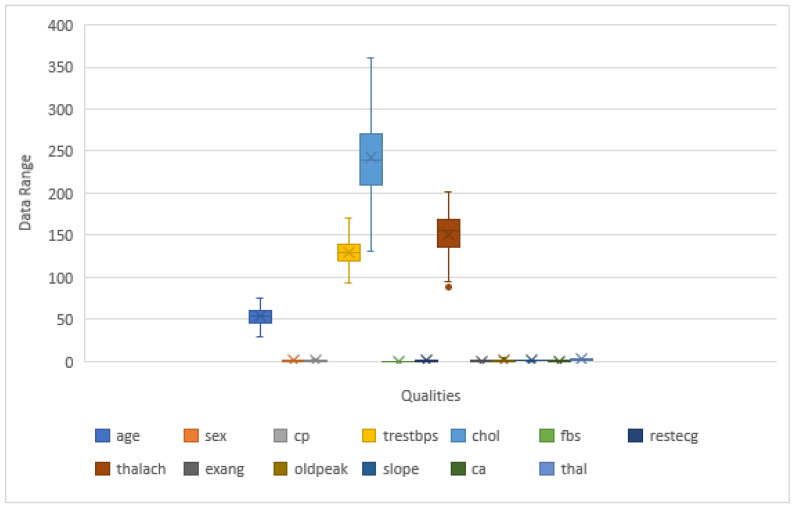
The changes of box plot after the outlier removal using IQR in the Cleveland dataset.

The dataset’s pattern determines the success of classification challenges. Falling values seldom hamper the result. Therefore, in the beginning, we examined the dataset to see whether it had any lost values or not. The mislaid values can be verified in various ways, including totally ignoring them, replacing them with any numeric value, replacing them with the maximum time resembling that property, or restoring the value with the mean value for that property. Cleveland, Hungary, Switzerland, and Long Beach (CHSLB) have no missing variables in the combined dataset. In addition, there are no missing values in the Cleveland dataset. Data preprocessing is the process of transforming raw data into an understandable format. The quality of the data should be checked before applying machine learning or data mining algorithms. There are many ways to process data; however, in this study, we considered the outlier detection method. The CHSLB dataset shows normal distribution, but the Cleveland dataset is not normally distributed. For outliers’ detection, we used the IQR method. This method is used when the data are not normally distributed. If data are skewed, the IQR method is suitable for data preprocessing. There are 4 methods for finding IQR, such as ordering the data from least to greatest, finding the median, calculating the median of both the lower and upper half of the data, and the IQR difference between the upper and lower medians. To calculate the minimum, we used (Q1 − 1.5×IQR), while (Q3 − 1.5 × IQR) was used for the calculation of the maximum, and these whole things are called IQR proximity roles. Here, Q1 is 25 percentiles, and Q3 is 75 percentiles, and IQR is a range of Q1 and Q3, which means the difference between 25 percentiles and 75 percentiles, such as (IQR = Q2 − Q1). At the end of this study, we used trimming. Figure 2 shows the box plot, which has whiskers and, outside the whisker, presents the value, which is called the outliers. Figure 3 shows the changes in the box plot after the outlier removal using IQR in the Cleveland dataset. Since the outliers scale back the performance of the model’s rules, this model is significant for this study. 

### 4.5. Classification Modeling

#### 4.5.1. Random Forest

Random forests organize decision trees on randomly selected information units, prepare a forecast per tree, and opt for the fittest answer through voting. It additionally offers a fairly smart pointer of the feature’s importance. This composite classifier produces varied decision trees and incorporates them to urge the foremost effective result. For tree learning, principally implements bootstrap aggregating or bagging.

#### 4.5.2. Decision Tree

The decision Tree formula applies to the family of supervised learning algorithms. In distinction to different supervised learning algorithms, the selection tree algorithms are used for locating regression and classification problems. The aim of using a choice tree is to vogue a training model, which can predict the class or advantage of the victim variable by learning easy decision rules induced from training data.

#### 4.5.3. Implementation of the Techniques by Using Two Datasets

The following section involves the specifications of each technique’s learning parameters.

##### Combined Cleveland, Hungary, Switzerland, and Long Beach Dataset:

For decision tree:

- Criterion: The function to measure the quality of a split supported criteria is “Gini” for the Gini impurity and “entropy” for the information gain. In this paper, the researcher used “entropy”.
- Splitter: The strategy used to choose the split at each node. Supported strategies are “best” to choose the best division and “random” to choose the best random split. In this study, the researcher used “random”.
- Max_features: The numbers of features are “auto”, “sqrt”, and “log2” to think about while deciding on the optimal split. This study used “auto”.

For random forest:

- Criterion: The function for determining a split’s quality. The Gini impurity is supported by the criterion “Gini”, while the criterion “entropy” is a tree-specific parameter. In this study, the researcher used “entropy”.
- Max_samples: The number of samples to draw from X to train the individual base estimator if bootstrap is valid. This study used max_samples = 710.

For AdaBoost algorithm:

- n_estimators: The number of estimators at which boosting is stopped. In a perfect match, the learning operation is terminated early. This study used n_estimators = 550.

For the KNN algorithm:

- Algorithm: The nearest neighbors were computed using an algorithm. We utilized the algorithm “auto” in this investigation.
- Auto: “Auto” tries to find the most appropriate set of rules that are solely on the values exceeded to suit the technique.
- N_jobs: The number of parallel jobs that must be executed to find neighbors. Unless in the context of joblib parallel backend, none indicates 1; –1 indicates that all processors are being used, which is available in the Glossary. The fit technique is unaffected, and this study used n_jobs is 1.
- N_neighbors: The default number of neighbors for K-neighbors queries. This study utilized n neighbors = 10.
- P: The Minkowski metric’s strength element. The *p* = 1 is identical to the use of Manhattan distance (l1), and *p* = 2 is comparable in using Euclidean distance (l2). Minkowski distance (l *p*) is utilized for arbitrary *p*. This study used *p* =1.
- Weights: This is the distance to measure and utilize the tree. Minkowski is the default metric, and it is identical to the normal Euclidean metric with *p* = 2. A list of possible metrics may be found in the distance metric documentation. X is considered to be a distance matrix and must be squared during fit if the metric is “precomputed”. Only nodes with “nonzero” values are considered neighbors if X is a sparse graph. This study utilized a weight to measure “distance” in this analysis.

##### Cleveland Dataset:

For random forest:

- Max_samples: The number of samples to draw from X to train each base estimator if bootstrap is true. This study used max_samples = 80.
- Criterion: The forest’s total amount of trees. For this study, the criterion is “entropy”.

For KNN:

- N_jobs: The number of parallel jobs must be executed to find neighbors. Unless in the context of joblib parallel backend, none indicates 1; −1 indicates that all processors are being used. The fit technique is unaffected, and for N, jobs are −1 in this study.
- P: The Minkowski metric’s strength parameter. When *p* = 1, this is identical to the use of Manhattan distance (l1), and when *p* = 2, this is comparable to the use of the Euclidean distance (l2). Minkowski distance (l *p*) is utilized for arbitrary *p*. In this study, the researcher considered *p* = 1.

For decision tree:

- Criterion: The feature is to a degree the exception of a split. Additionally, supported standards are “Gini” for the Gini impurity and “entropy” for the data gain. This parameter is tree-specific. In this study, entropy was used.

For AdaBoost algorithm:

- n_estimators: The number of estimators at which boosting is stopped. In the event of a perfect match, the learning operation is terminated early. This study used n estimators = 450.

## 5. Results and Discussion

In this paper, four machine learning algorithms, such as RF, AB, DT, and KNN, were used for both Cleveland, Hungary, Switzerland, and Long Beach (CHSLB) and Cleveland datasets. A total of 1025 samples were extracted from the CHSLB database. There are two sorts of diagnoses: normal and patients at risk of heart disease. Among the 1025 samples, 499 showed no evidence of heart illness, and 526 showed evidence of heart disease. Among 303 data in the Cleveland dataset, 138 show the absence of heart disease, and 165 identify the presence of heart disease. The confusion metrics for evaluating the heart disease detection system of test data using CHSLB and Cleveland in our study are given in Table 2 and Table 3. 

**Figure 4:**
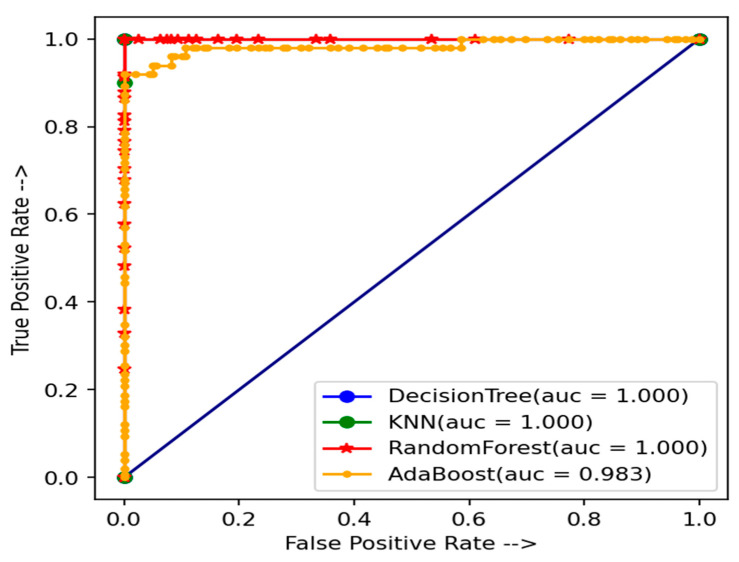
The AUC curve of test data using the CHSLB datasets for the used models.

**Figure 5:**
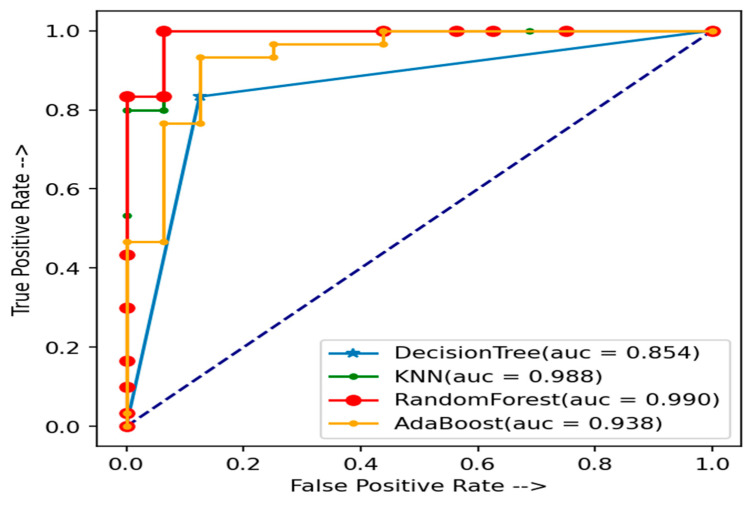
The AUC curve of test data using the Cleveland dataset for the used models.

The AUC curve shows the effects of evaluating the heart disease detection system. Figure 4 and Figure 5 show the effects of the AUC curve using the test data of CHSLB and Cleveland, respectively. From the AUC curve, it is clear that our proposed model performed better to measure the accuracy for predicting heart disease from our used datasets.

The performance matrices of CHSLB and Cleveland datasets for different used models for evaluating the heart disease detection system are given in Table 4 and Table 5, respectively. Table 4 shows accuracies of 99.03%, 96.11%, 100%, and 100% by utilizing RF, AB, DT, and KNN, respectively. Further, the additional performance assessment parameters such as precision, recall, f1-score, MAE, and R^2^ score are shown in the same table. This study found a performance of 1.00 for precision (1) and recall (0) of all models where other parameters have been changed slightly. On the other hand, all changes in performance parameters corresponding to the used model for Cleveland datasets are shown in Table 5.

The obtained accuracies for the used models in this study and other existing models are compared in Table 6. This study found the highest result for the CHSLB datasets compared to the literature [65,66,67,68,69,70,71,72,73,74]. Moreover, most of the results given in related works in Section 2 [2,48,49,50,51,52] are less significant than the proposed models. 

**Figure 6:**
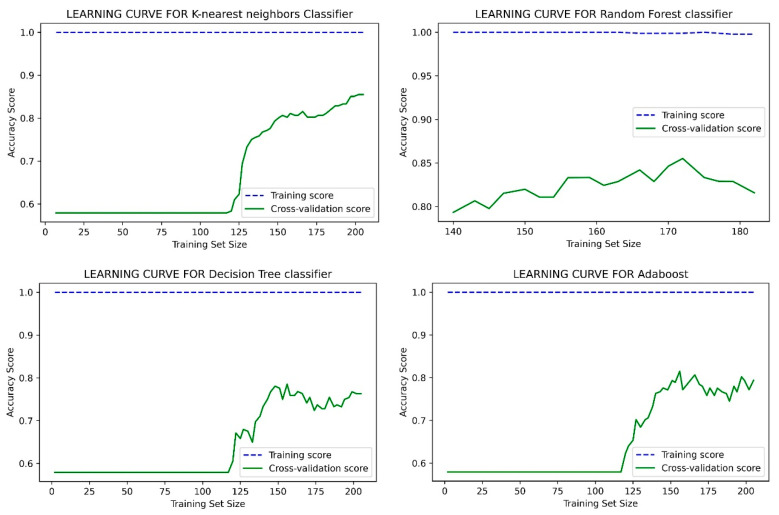
The accuracy performance graph for the Cleveland dataset.

**Figure 7:**
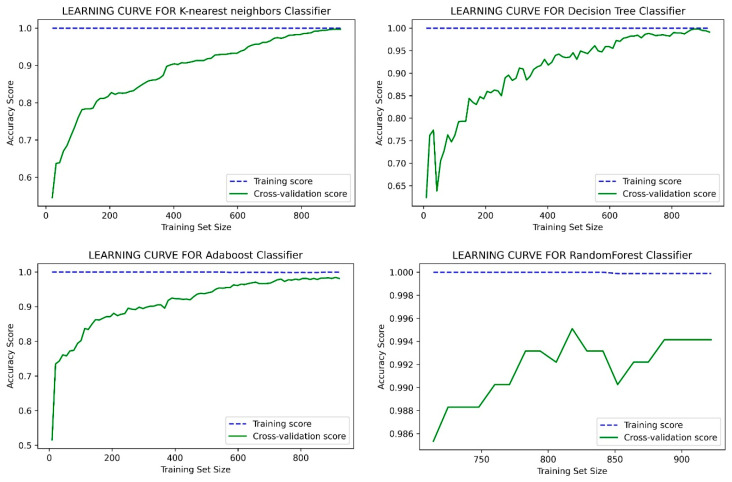
The accuracy performance graph for the CHSLB datasets.

This study has obtained better accuracy than the results reported in the references [2,65,66,67,68,69,70,71,72,73,74]. In those studies, the authors suggested introducing an expert system to improve the prediction accuracy. Like this study, the authors in ref. [48] also introduced an intelligence system, namely NN-based prediction of CHD risk using feature correlation analysis (NN-FCA). In [52], the authors used a reliable feature selection method for HD disease prediction by using a minimal number of attributes instead of considering all available attributes. In [65], the accuracy was obtained by stacking ensembles selection with threshold features. In refs. [2,66,74], the authors did not perform any pre-filtering and trimming of data to fit the model better. In [66], the authors did not mention their model’s tuning parameters; ref. [67], did not show any specific data cleaning methodology, and their training model parameters are also not mentioned. In the work [69], the authors’ extracted unstructured data manually through a cardiologist, and such a technique is not possible for online public datasets. In the work [72], the authors’ mentioned the feature selection, but the total number of features for the Cleveland dataset is already low. Another feature selection might create a classification bias. In our paper, so far, we performed pre-filtering and trimming to fit the model better. Along with this, we also adopted a range of hyper-parameters (as explained in the earlier section) and the training setup to train the model more perfectly. It is assumed that our adopted technique helped to obtain better accuracy in this study. On the other hand, different datasets were used by other studies, such as the Armed forces institute of cardiology [68], Kita Hospital Jakarta (450) [70], People’s Hospital dataset [72], and Northern Lebanon [73], and all show poor accuracy. The accuracy performance graph of our proposed model is given in Figure 6 and Figure 7 for the Cleveland and CHSLB datasets, respectively.

**Figure 8:**
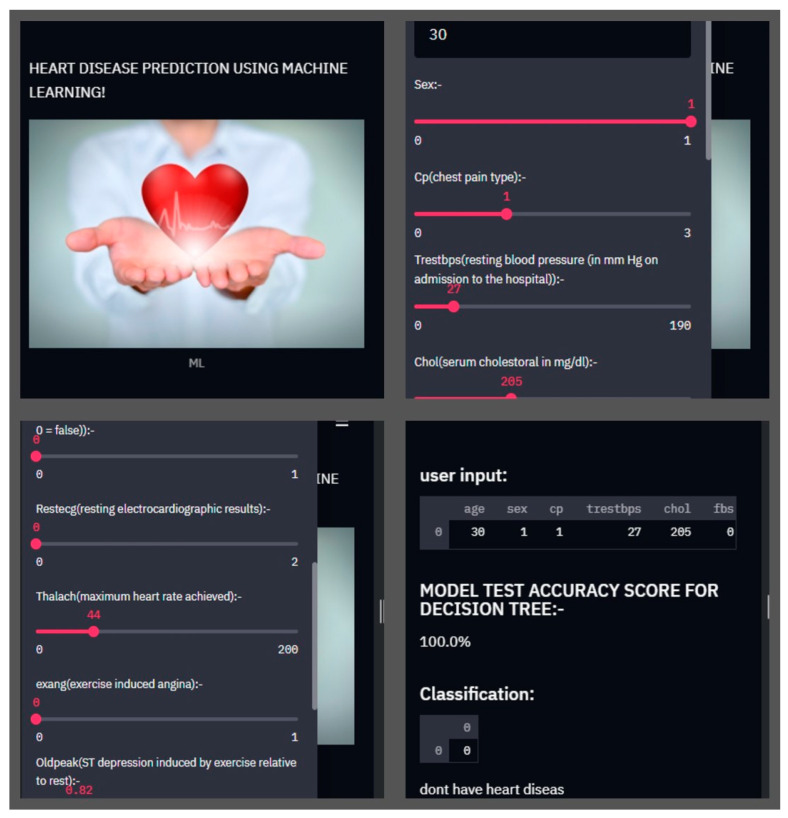
Real-time web-based smart system for heart disease prediction.

Finally, this study used an internet app and Streamlit cloud hosting to anticipate the sickness of CHD. The webpage link for our proposed system is https://share.streamlit.io/emonkumardas/heart.github.io/main/heart.py (accessed on 13 June 2022). The attribute values acquired from patients are transferred to a cloud server, where the constructed model is stored using a web server and a web application. Patients and doctors receive the forecast via the cloud server. Figure 8 depicts the implementation duration of the system’s coronary cardiovascular disease prediction method. For various input attribute values, the mobile application displays the expected result. This application will be used by both the patient and the doctor for their respective purposes. To begin, patients have to open the app and enter some attributes, such as age, sex, chest pain kind, blood pressure, etc. The input values are sent to a web server, where they are saved. The anticipated model is placed on the cloud server, and the result is projected using the value of the attribute and then sent back to the webserver. This outcome is likewise saved on the internet server. Patients and doctors should continue observing to see if the expected result of cardiovascular disease is active or not. We used the CHSLB and Cleveland datasets in this web tool, and the most effective models provided 100 percent and approximately 97 percent correct results. 

## 6. Conclusions

Heart disease is challenging, and it kills thousands of people each year. If the initial signs of heart disease are neglected, the patient may have substantial repercussions in a concise period. This study employed four machine learning models (RF, DT, AB, and KNN) to predict coronary heart disease using CHSLB (Cleveland, Hungary, Switzerland, and Long Beach) and Cleveland datasets. The data were preprocessed using some appropriate methods and techniques in order to improve the detection accuracy of the used ML models. Among the studied models, the KNN shows a better accuracy of 100% and 97.82% with the CHSLB and Cleveland datasets, respectively. In the case of the CHSLB dataset, RF, AB, and DT models show relatively better accuracy of 99.025%, 96.103%, and 100%, respectively. This type of process intelligence approach is critical in medical diagnosis. Following the improved detection accuracy of the used ML algorithms, a computer-aided smart system together with the freely accessible internet-based cloud hosting platform was developed. It is expected that the developed system will assist in the diagnosis of cardiac problems in a very convenient manner, i.e., making the doctor’s job simpler. Above all, the study has made a significant addition to the computation of strength ratings that are strong predictors of heart disease prognosis.

The applied process can be improved by adding more data, doing k-fold cross-validation, checking for overfitting issues, and testing with more critical or statistically generated data such as numeric data augmentation. The authors consider this to be an upgradable future work. 

## Figures and Tables

**Table 1:** One database has four datasets that connect Cleveland, Hungary, Switzerland, and Long Beach (CHSL), while the other contains a dataset from the Cleveland heart disease dataset. Both databases are described in detail below.

Si. No.	Qualities	Variety	Standard
(i)	Age	Integer	29–77
(ii)	Sex	Integer	male = 1; female = 0
(iii)	Chest pain type	Integer	angina = 0; abnanr = 1;
			notang = 2;
			asympt = 3
(iv)	Blood pressure value	Integer	94–200
(v)	Serum cholesterol	Integer	126–564
(vi)	Fasting blood sugar	Integer	true = 1; false = 0
(vii)	Resting electro-cardiographic results	Integer	0–2
(viii)	Maximum heart rate	Integer	71–202
(x)	Old peak	Float	0.0–6.2
(xi)	The slant of the peak exercise ST segment	Integer	upsloping = 0; flat = 1;
			Down sloping = 2
(xii)	Number of major vessels	Integer	0–4
	Exercise-induced angina	integer	1 = yes; 0 = no
(xiii)	Thal	Integer	
			defect = 6; reversible
			defect = 7
(xiv)	Coronary heart disease	Integer	present = 1; absent = 0

**Table 2:** The confusion metrics for evaluating the heart disease detection system of test data using Cleveland, Hungary, Switzerland, and Long Beach (CHSLB) dataset for the used models.

Sr. No.	Used Model for CHSLB Datasets	Predicted Value (Actual Class)	Predicted Value			Actual Value
1.	Random Forest	N = 308		NO	YES	
			NO	TN = 159	*FP* = 0	159
			YES	*FN* = 3	*TP* = 146	149
			Total predict	162	146	308
2.	AdaBoost	N = 308		NO	YES	
			NO	*TN* = 159	*FP* = 0	159
			YES	*FN* = 12	*TP* = 137	149
			Total predict	171	137	308
3.	Decision Tree	N = 308		NO	YES	
			NO	*TN* = 159	*FP* = 0	159
			YES	*FN* = 0	*TP* = 149	149
			Total predict	159	149	308
4.	KNN	N = 308		NO	YES	
			NO	*TN* = 159	*FP* = 0	159
			YES	*FN* = 0	*TP* = 149	149
			Total predict	159	149	308

**Table 3:** The confusion metrics for evaluating the heart disease detection system of test data using the Cleveland dataset for the used models.

Sr. No.	Used Model for Cleveland Datasets	Predicted Value (Actual Class)	Predicted Value			Actual Value
1.	Random Forest	N = 46		NO	YES	
			NO	*TN* = 15	*FP* = 1	16
			YES	*FN* = 1	*TP* = 29	30
			Total predict	16	30	46
2.	AdaBoost	N = 46		NO	YES	
			NO	*TN* = 14	*FP* = 2	16
			YES	*FN* = 2	*TP* = 28	30
			Total predict	16	30	46
3.	Decision Tree	N = 46		NO	YES	
			NO	*TN* = 13	*FP* = 3	16
			YES	*FN* = 10	*TP* = 20	30
			Total predict	23	23	46
4.	KNN	N = 46		NO	YES	
			NO	*TN* = 15	*FP* = 1	16
			YES	*FN* = 0	*TP* = 30	30
			Total predict	15	31	46

**Table 4:** Performances matrices for evaluating the heart disease detection system of CHSLB datasets for used models.

Performance Matrices	Models			
	RF	AB	DT	KNN
Accuracy	99.03%	96.10%	100%	100%
Precision (0)	0.98	0.93	1.00	1.00
Precision (1)	1.00	1.00	1.00	1.00
Recall (0)	1.00	1.00	1.00	1.00
Recall (1)	0.98	0.92	1.00	1.00
F1-score (0)	0.99	0.96	1.00	1.00
F1-score (1)	0.99	0.96	1.00	1.00
MAE	0.00974	0.0389610	0.0	0.0
R^2^ Score	96.09	84.08	1.0	1.0

**Table 5:** Performances matrices for evaluating the heart disease detection system of Cleveland dataset for used models.

Performance Matrices	Models			
	RF	AB	DT	KNN
accuracy	93.478%	91.30%	71.739%	97.826%
Precision (0)	0.88	0.88	0.57	1.00
Precision (1)	0.97	0.93	0.87	0.97
Recall (0)	0.94	0.88	0.81	0.94
recall (1)	0.93	0.93	0.67	1.00
F1-score (0)	0.91	0.88	0.67	0.97
f1-score (1)	0.95	0.93	0.75	0.98
MAE	6.521%	8.69	28.260%	2.173%
R^2^ Score	71.249%	61.66%	71.249%	90.41%

**Table 6:** A comparison of the proposed system’s accuracy with the existing results.

Sr. No.	Used Data Set	Models			
		RF	AB	DT	KNN
1	CHSLB datasets (1025) (Present study)	99.03%	96.10%	100%	100%
	Cleveland dataset (303) (Present study)	93.478%	91.30%	71.739%	97.826%
2	Five-fold in the statlog dataset	90.46% [2]	-	96.42% [2]	96.42% [2]
3.	Cleveland dataset (303)			75.55% [65]	90.16% [66]
4.	Cleveland dataset (303)				80% [67]
5.	Armed forces institute of cardiology	68.6% [68]		86.6% [68]	
6.	CHSLB datasets (920)	80.89% [69]			
7.	Kita Hospital Jakarta (450)		46% [70]		
8.	Cleveland dataset (303)		54.13% [71]		
9.	Cleveland dataset (303)	91.6% [72]			
10	People’s Hospital dataset	97% [72]			
11	Northern Lebanon	97.7% [73]			
12	Cleveland dataset (303)	84% [74]	-	79% [74]	87% [74]

## Data Availability

All data are available in the manuscript.
